# Different Profile of mRNA Expression in Sinoatrial Node from Streptozotocin-Induced Diabetic Rat

**DOI:** 10.1371/journal.pone.0153934

**Published:** 2016-04-20

**Authors:** Zannatul Ferdous, Muhammad Anwar Qureshi, Petrilla Jayaprakash, Khatija Parekh, Annie John, Murat Oz, Haider Raza, Halina Dobrzynski, Thomas Edward Adrian, Frank Christopher Howarth

**Affiliations:** 1 Department of Physiology, College of Medicine & Health Sciences, UAE University, Al Ain, UAE; 2 Department of Biochemistry, College of Medicine & Health Sciences, UAE University, Al Ain, UAE; 3 Department of Pharmacology, College of Medicine & Health Sciences, UAE University, Al AIn, UAE; 4 Institute of Cardiovascular Sciences, University of Manchester, Manchester, United Kingdom; New York Medical College, UNITED STATES

## Abstract

**Background:**

Experiments in isolated perfused heart have shown that heart rate is lower and sinoatrial node (SAN) action potential duration is longer in streptozotocin (STZ)–induced diabetic rat compared to controls. In sino-atrial preparations the pacemaker cycle length and sino-atrial conduction time are prolonged in STZ heart. To further clarify the molecular basis of electrical disturbances in the diabetic heart the profile of mRNA encoding a wide variety of proteins associated with the generation and transmission of electrical activity has been evaluated in the SAN of STZ-induced diabetic rat heart.

**Methodology/Principal Findings:**

Heart rate was measured in isolated perfused heart with an extracellular suction electrode. Expression of mRNA encoding a variety of intercellular proteins, intracellular Ca^2+^-transport and regulatory proteins, cell membrane transport proteins and calcium, sodium and potassium channel proteins were measured in SAN and right atrial (RA) biopsies using real-time reverse transcription polymerase chain reaction techniques. Heart rate was lower in STZ (203±7 bpm) compared to control (239±11 bpm) rat. Among many differences in the profile of mRNA there are some worthy of particular emphasis. Expression of genes encoding some proteins were significantly downregulated in STZ-SAN: calcium channel, *Cacng4* (7-fold); potassium channel, *Kcnd2* whilst genes encoding some other proteins were significantly upregulated in STZ-SAN: gap junction, *Gjc1*; cell membrane transport, *Slc8a1*, *Trpc1*, *Trpc6* (4-fold); intracellular Ca^2+^-transport, *Ryr3*; calcium channel *Cacna1g*, *Cacna1h*, *Cacnb3*; potassium channels, *Kcnj5*, *Kcnk3* and natriuretic peptides, *Nppa* (5-fold) and *Nppb* (7-fold).

**Conclusions/Significance:**

Collectively, this study has demonstrated differences in the profile of mRNA encoding a variety of proteins that are associated with the generation, conduction and regulation of electrical signals in the SAN of STZ-induced diabetic rat heart. Data from this study will provide a basis for a substantial range of future studies to investigate whether these changes in mRNA translate into changes in electrophysiological function.

## Introduction

Cardiovascular complications are common in patients with Type 1 and Type 2 diabetes mellitus and these complications lead to an increased risk of mortality [1]. Although vascular diseases including coronary artery disease and hypertension increase the risk of mortality these patients are also at increased risk of developing cardiac abnormalities that are independent of cardiovascular complications [2]. In addition to mechanical dysfunction disturbances in electrical activity, manifesting as arrhythmias, have also been widely reported in diabetic heart. Patients with diabetes have an increased risk of arrhythmias including QT interval and QRS prolongation which is associated with sudden death [3] Atrial fibrillation, bradyarrhythmias, bundle branch block and atrioventricular block are more prevalent in diabetic patients [4]. *In vivo* biotelemetry studies performed in experimental models of diabetes have demonstrated disturbances in the electrocardiogram which are variously associated with bradycardia, prolongation of PQ, QRS and QT intervals [5–7]. Action potential duration is frequently prolonged in the diabetic heart and prolongation can occur to different extents in different regions of the heart including the SAN [6,8,9]. Very little experimental data is available about the effects of diabetes on SAN electrophysiology. It is known that SAN conduction and pacemaker cycle length are prolonged and that the SAN action potential duration may be prolonged in diabetic heart [10,11]. A variety of ionic currents including L-type and T-type Ca^2+^ current, hyperpolarization-activated "funny" current, Na^+^/Ca^2+^ exchange current and various K^+^ currents are important in the generation of the SAN action potential [12]. Sarcoplasmic reticulum (SR) Ca^2+^ signaling may also contribute to the generation and decay of the SAN action potential [12]. Disturbances of one or more of these ionic conductances would undoubtedly have implications for the generation and conduction of electrical signals in the SAN which in turn may underlie some of the electrical disturbances that have been frequently reported in diabetic heart [5–7]. To further clarify the molecular basis of electrical disturbances in the SAN of diabetic heart the profile of mRNA that encodes a wide variety of proteins that are associated with the generation and conduction of electrical activity in the pacemaker has been evaluated in the STZ-induced diabetic rat heart.

## Materials and Methods

### Experimental protocol

Forty male Wistar rats aged 8 weeks were divided into 2 subgroups. All animals received normal rat chow and drinking water *ad libitum*. One subgroup of rats received STZ/citrate buffer (60 mg/kg, intraperitoneal) whilst the other subgroup received citrate buffer alone. Experiments began 10 weeks after STZ treatment. Blood glucose was measured 5 days following STZ treatment to confirm diabetes. Body weight, heart weight and blood glucose were measured immediately prior to experiments. All animal experimentation was carried out in accordance with the Animals (Scientific Procedures) Act 1986 and conforms to the Recommendation from the Declaration of Helsinki and the Guiding Principles in the Care and Use of Animals. Approval for this project was obtained from the Animal Ethics Committee, College of Medicine & Health Sciences, United Arab Emirates University.

### Measurement of heart rate and action potentials

Rats were killed using a guillotine. Hearts were then rapidly removed, mounted in Langendorff mode and perfused retrogradely at a constant flow of 8 ml.g heart^-1^ min^-1^ and at physiological temperature (36–37°C) with a normal Tyrode containing: 140 mM NaCl; 5 mM KCl; 1 mM MgCl_2_; 10 mM glucose; 5 mM HEPES; 1.8 mM CaCl_2_ and adjusted to pH 7.4 with NaOH and continuously bubbled with oxygen. To measure heart rate action potentials were recorded in spontaneously beating hearts with a purpose built extracellular suction electrode with a tip ~ 2 mm in diameter according to previously described techniques [13]. Recordings were made in the region of the left ventricle. Signals from the electrode were collected at 400 Hz, amplified (ADInstuments, ML136 Bioamp) and conveyed via a Powerlab (ADInstruments, PL410) for display on a PC. Data were analyzed with ADInstruments software version v 4.21 (ADInstruments, Australia).

### Expression of mRNA

**Fig 1:**
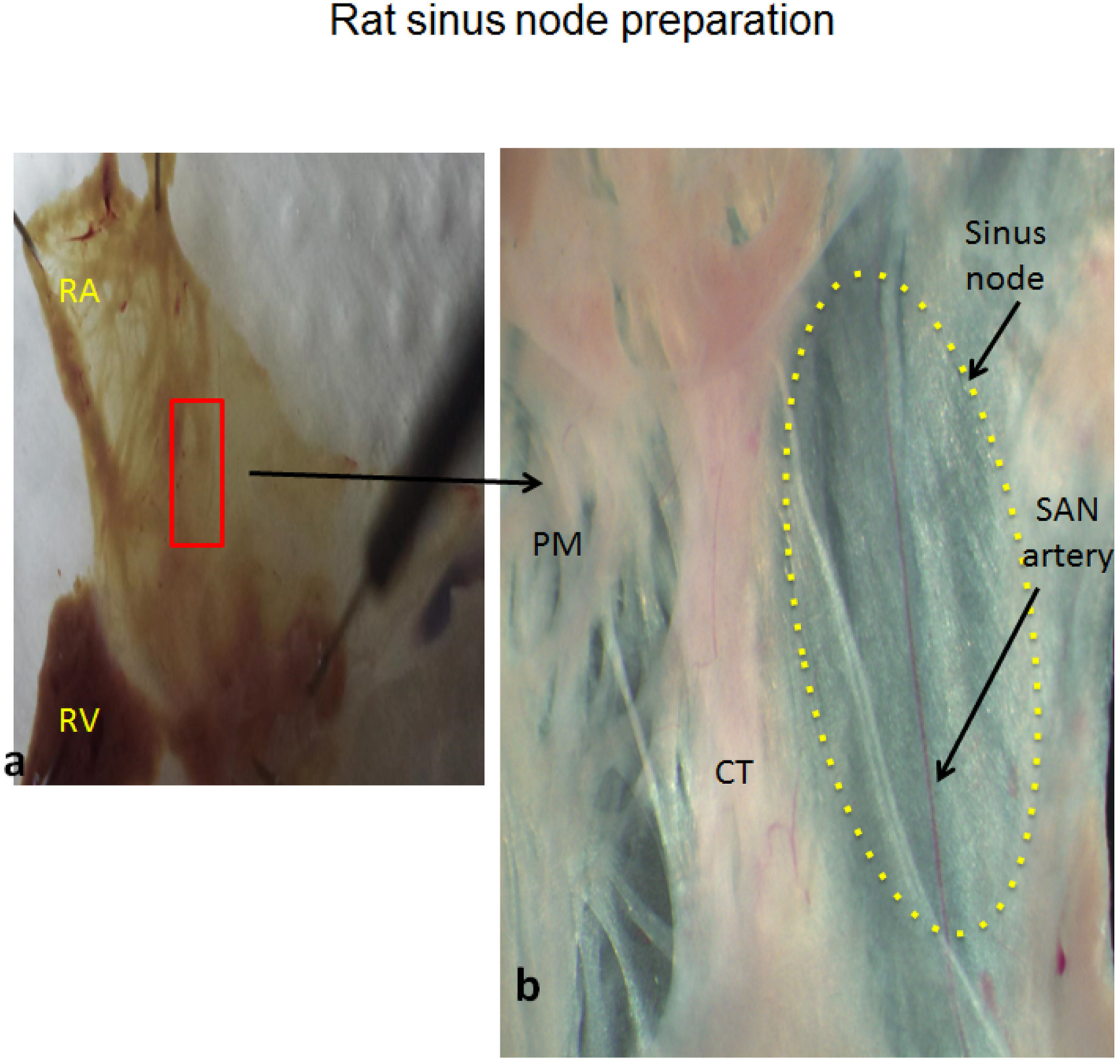
a & b –Gross anatomy of the sinus node in a typical control heart showing the location where tissue samples were collected.

Expression of genes encoding a range of cardiac muscle proteins was assessed using modifications of previously described techniques [14–16]. After sacrifice hearts were removed rapidly from the rats and placed in a dish containing: NaCl 140 mM; KCl 5.4 mM; MgCl_2_ 1 mM; HEPES 5 mM; D-glucose 5.5 mM; CaCl_2_ 1.8 mM and adjusted to pH 7.4 with NaOH. The ventricles and the left atrium were removed and the right atrium was opened to expose the SAN and crista terminalis (Fig 1a and 1b). The SAN artery was used to identify the SAN. The SAN was exposed and 2 mm biopsy samples of SAN were carefully collected from 20 STZ (STZ-SAN) and 20 control (CON-SAN) hearts according to previously described techniques [17,18]. Samples of right atrial (RA) tissue were also collected. Once dissected, the samples were immediately placed in RNAlater (AM7021, Life Technologies, Carlsbad, CA, USA) and kept overnight at room temperature to allow thorough penetration of the tissue. Tissue samples were then frozen at -20°C pending further processing. The samples were homogenized at 6500 rpm for 2 runs of 20 seconds each with a 15 second gap (Preceylls 24, Berlin Technology, USA). Isolation of total RNA from the tissue was performed using the SV Total RNA Isolation System (Promega, Madison, USA) according to the manufacturer’s instructions. The concentration and purity of the RNA samples was determined by measuring the absorbance at 260 nm and the ratio of absorbance at 260 nm and 280 nm (ND-1000, NanoDrop). A two-step RT-PCR procedure was used to generate cDNA. Total RNA (500 ng) was converted into cDNA in a 25 μl PCR reaction with 10 x RT Buffer 2.0 μl, 25 x dNTP Mix (100 mM) 0.8 μl, 10 x RT Random Primers 2.0 μl, MultiScribe^™^ Reverse Transcriptase 1.0 μl, RNase inhibitor 1.0 μl, and Nuclease-free H_2_O (High Capacity cDNA Reverse Transcription Kit (4374966, Applied Biosystems, USA). Reverse transcription was carried out using the following parameter values: 25°C for 10 min, 37°C for 120 min, and 85°C for 5 min on the Veriti thermal cycler (Applied Biosystems, USA). Gene Expression Assays were performed using custom TaqMan Low Density Arrays (Format 32, 4346799, Applied Biosystems, USA). The TaqMan assays are pre-loaded in each reaction well of the array in triplicate for each RNA sample. As in previous studies 18S ribosomal RNA was used as an endogenous control [19]. Expression of 18S was not significantly different (P>0.05) between the samples of STZ-SAN, CON-SAN, STZ-RA and CON-RA. 100 ng of cDNA (RNA-equivalent) was loaded together with 2 × TaqMan Gene Expression Master Mix (No AmpErase UNG, Applied Biosystems, USA) for a total of 100 μL per port. Two SAN samples were combined and two RA samples were combined for each real-time RT-PCR assay. Real-time RT-PCR was performed in a Fast ABI Prism 7900HT Sequence Detection System (Applied Biosystems, USA). The PCR thermal cycling parameters were run in standard mode as follows: 50°C for 2 min, 94.5°C for 10 min, followed by 40 cycles of 97°C for 30 sec and 59.7 for 1 min. Results were initially analyzed using ABI Prism 7900HT SDS, v2.4. All remaining calculations and statistical analysis were performed by the SDS RQ Manager 1.1.4 software using the 2−ΔΔCt method with a relative quantification RQmin/RQmax confidence set at 95%. A list of the target genes and the proteins encoded by these genes is shown in Table 1.

**Table 1:** Target genes and proteins.

Genes	Proteins	Protein descriptions
Cardiac muscle and associated regulatory proteins		
*Myh6*	MHC-α	Myosin Heavy Chain, Cardiac Muscle Alpha Isoform
*Myl1*	MLC1	Myosin light chain 3, skeletal muscle isoform
*Myl2*	MLC-2	Myosin regulatory light chain 2, ventricular/cardiac muscle isoform
*Tnnc1*	TN-C	Troponin C Type 1
**Intercellular proteins**		
*Gja1*	Cx43	Connexin43
*Gja5*	Cx40	Connexin40
*Gja7*	Cx45	Connexin45
*Gjd3*	Cx31.9	Connexin31.9
**Cell membrane transport**		
*Atp1a1*	Na/K ATPase,α1	ATPase, Na+/K+ Transporting, Alpha 1 Polypeptide
*Atp1a2*	Na/K ATPase,α2	ATPase, Na+/K+ Transporting, Alpha 2 Polypeptide
*Atp1a3*	Na/K ATPase,α3	ATPase, Na+/K+ Transporting, Alpha 3 Polypeptide
*Atp1b1*	Na/K ATPase,β1	ATPase, Na+/K+ Transporting, Beta 1 Polypeptide
*Atp2b1*	Na/K ATPase,β2	ATPase, Ca++ Transporting, Plasma Membrane 1
*Slc8a1*	NCX1	Solute Carrier Family 8 (Sodium/Calcium Exchanger), Member 1
*Trpc1*	TRPC1	Transient receptor potential channel 1
*Trpc3*	TRPC3	Transient receptor potential channel 3
*Trpc6*	TRPC6	Transient receptor potential channel 6
**Intercellular Ca**^**2+**^ **transport and Ca**^**2+**^ **regulation**		
*Atp2a2*	SERCA2	Sarcoplasmic/Endoplasmic Reticulum Calcium ATPase 2
*Calm1*	Calm1	Calmodulin1
*Calm3*	Calm3	Calmodulin3
*Casq2*	**Casq2**	Calsequestrin 2
*Itpr1*	IP3R1	Inositol 1,4,5-Trisphosphate Receptor, Type 1
*Itpr2*	IP3R2	Inositol 1,4,5-Trisphosphate Receptor, Type 2
*Itpr3*	IP3R3	Inositol 1,4,5-Trisphosphate Receptor, Type 3
*Pln*	PLB	Phospholamban
*Ryr2/* RYR2	RYR2	Ryanodine Receptor 2
*Ryr3/* RYR3	RYR3	Ryanodine Receptor 3
**Hyperpolarization-activated cyclic nucleotide-gated channels**		
*Hcn1*	HCN1	Hyperpolarization-activated cyclic nucleotide-gated channels 1
*Hcn2*	HCN2	Hyperpolarization-activated cyclic nucleotide-gated channels 2
*Hcn3*	HCN3	Hyperpolarization-activated cyclic nucleotide-gated channels 3
*Hcn4*	**HCN4**	Hyperpolarization-activated cyclic nucleotide-gated channels 4
**Calcium channels**		
*Cacna1c*	Ca_v_1.2	Voltage-Dependent, L Type, Alpha 1C Subunit
*Cacna1d*	Ca_v_1.3	Voltage-Dependent, L Type, Alpha 1D Subunit
*Cacna1g*	Ca_v_3.1	Voltage-Dependent, T Type, Alpha 1G Subunit
*Cacna1h*	Ca_v_3.2	Voltage-Dependent, T Type, Alpha 1H Subunit
*Cacna2d1*	Ca_v_α2δ1	Voltage-Dependent, Alpha 2/Delta Subunit 1
*Cacna2d2*	Ca_v_α2δ2	Voltage-Dependent, Alpha 2/Delta Subunit 2
*Cacna2d3*	Ca_v_α2δ3	Voltage-Dependent, Alpha 2/Delta Subunit 3
*Cacnb1*	Ca_v_β1	Voltage-Dependent, Beta 1 Subunit
*Cacnb2*	Ca_v_β2	Voltage-Dependent, Beta 2 Subunit
*Cacnb3*	Ca_v_β3	Voltage-Dependent, Beta 3 Subunit
*Cacng4*	Ca_v_γ4	Voltage-Dependent, Gamma Subunit 4
*Cacng7*	Ca_v_γ7	Voltage-Dependent, Gamma Subunit 7
**Sodium channels**		
*Scn1a*	Na_v_1.1	Voltage Gated, Type I Alpha Subunit
*Scn1b*	Na_v_β1	Voltage Gated, Type I Beta Subunit
*Scn2b*	Na_v_β2	Voltage Gated, Type II Beta Subunit
*Scn3a*	Na_v_1.3	Voltage Gated, Type III Alpha Subunit
*Scn3b*	Na_v_β3	Voltage Gated, Type III Beta Subunit
*Scn4a*	Na_v_1.4	Voltage Gated, Type IV Alpha Subunit
*Scn5a*	Na_v_1.5	Voltage Gated, Type V Alpha Subunit
*Scn7a*	Na_v_2.1	Voltage Gated, Type VII Alpha Subunit
**Potassium channels**		
*Kcna2*	K_v_1.2	Voltage Gated Shaker Related Subfamily A, Member 2
*Kcna3*	K_v_1.3	Voltage Gated Shaker Related Subfamily A, Member 3
*Kcna4*	K_v_1.4	Voltage Gated Shaker Related Subfamily A, Member 4
*Kcna5*	K_v_1.5	Voltage Gated Shaker Related Subfamily A, Member 5
*Kcna6*	K_v_1.6	Voltage Gated Shaker Related Subfamily A, Member 6
*Kcnb1*	K_v_2.1	Voltage Gated Shab Related Subfamily B, Member 1
*Kcnd1*	K_v_4.1	Voltage Gated Shal Related Subfamily D, Member 1
*Kcnd2*	K_v_4.2	Voltage Gated Shal Related Subfamily D, Member 2
*Kcnd3*	K_v_4.3	Voltage Gated Shal Related Subfamily D, Member 3
*Kcne4*	MIRP3	Minimum Potassium Ion Channel-Related Peptide 3
*Kcnh2*	ERG-1	Ether-A-Go-Go-Related Protein 1
*Kcnip2*	KChIP2	Kv Channel Interacting Protein 2
*Kcnj11*	K_ir_6.2	Inwardly Rectifying Subfamily J, Member 11
*Kcnj12*	K_ir_2.2	Inwardly Rectifying Subfamily J, Member 12
*Kcnj14*	K_ir_2.4	Inwardly Rectifying Subfamily J, Member 14
*Kcnj2*	K_ir_2.1	Inwardly Rectifying Subfamily J, Member 2
*Kcnj3*	K_ir_3.1	Inwardly Rectifying Subfamily J, Member 3
*Kcnj5*	K_ir_3.4	Inwardly Rectifying Subfamily J, Member 5
*Kcnj8*	K_ir_6.1	Inwardly Rectifying Subfamily J, Member 8
*Kcnk1*	TWIK1	Two Pore Domain Subfamily K, Member 1
*Kcnk2*	TREK1	Two Pore Domain Subfamily K, Member 2
*Kcnk3*	K_2P_3.1	Two Pore Domain Subfamily K, Member 3
*Kcnk5*	K_2P_5.1	Two Pore Domain Subfamily K, Member 5
*Kcnk6*	TWIK2	Two Pore Domain Subfamily K, Member 6
*Kcnn1*	SK1	Calcium Activated Intermediate/Small Conductance Subfamily N Alpha, Member 1
*Kcnn2*	SK2	Calcium Activated Intermediate/Small Conductance Subfamily N Alpha, Member 2
*Kcnn3*	SK3	Calcium Activated Intermediate/Small Conductance Subfamily N Alpha, Member 3
*Kcnq1*	K_v_7.1	Voltage Gated KQT-Like Subfamily Q, Member 1
**Miscellaneous proteins**		
*Abcc8*	SUR1	ATP-binding cassette transporter sub-family C member 8
*Abcc9*	SUR2	ATP-binding cassette, sub-family C member 9
*Nppa*	ANP	Atrial natriuretic peptide
*Nppb*	BNP	Brain natriuretic peptide
*Pias3*	KChAP	Protein Inhibitor of activated STAT, 3

### Expression of protein

Protein expression was measured using previously described SDS-PAGE and Western blotting techniques [20]. SAN from STZ and control rats were dissected, rinsed with ice-cold saline and homogenised in 100 mM potassium phosphate buffer (pH7.4) containing 1 mM EDTA and 0.1mM phenylmethylsulfonyl fluoride at 6500 rpm for 2 runs of 20 seconds each with a 15 second gap (Preceylls 24 homogeniser, Berlin Technology, USA). Protein concentration was measured using Bio-Rad reagent. The supernatant was used for SDS-PAGE and Western Blotting. Briefly, 10–20 ug protein was electrophoretically separated onto 7.5% or 12% (depending on the molecular weight of the protein to be separated) polyacrylamide gels and transferred onto nitrocellulose membranes. The expression of the specific proteins was checked by immunoreaction with their specific antibodies by Western blot analysis. β-actin was used as a loading control. The blots were developed using the Pierce Western Blot kit. Densitometric analysis of the protein bands was performed using the Typhoon FLA 9500, GE Healthcare Bio-Sciences AB (Uppsala, Sweden). The ratio of specific protein signal to that of actin control were used to calculate fold change.

### Statistics

Results were expressed as the mean ± S.E.M. of ‘n’ observations. Statistical comparisons were performed using one-way ANOVA and Bonferroni post hoc for multiple comparisons or Independent Samples t-test, as appropriate (SPSS v. 20). P< 0.05 was considered to indicate a significant difference.

## Results

Body and heart weight were significantly (P<0.01) reduced and heart weight / body weight ratio was increased in STZ compared to control rat. Blood glucose was increased 5-fold in STZ rat compared to control (Table 2).

**Table 2:** General characteristics of streptozotocin-induced diabetic rats.

	Control	Streptozotocin
Body weight (g)	322.50±22.98	207.00±51.99**
Heart weight (g)	1.15±0.12	0.85±0.13**
Heart weight / Body weight ratio	3.59±0.45	4.19±0.48**
Blood glucose (mg/dl)	96.92±15.03	514.33±54.60**

Data are mean ± SEM, n = 12 hearts,

** P<0.01

### Heart rate and action potential

**Fig 2:**
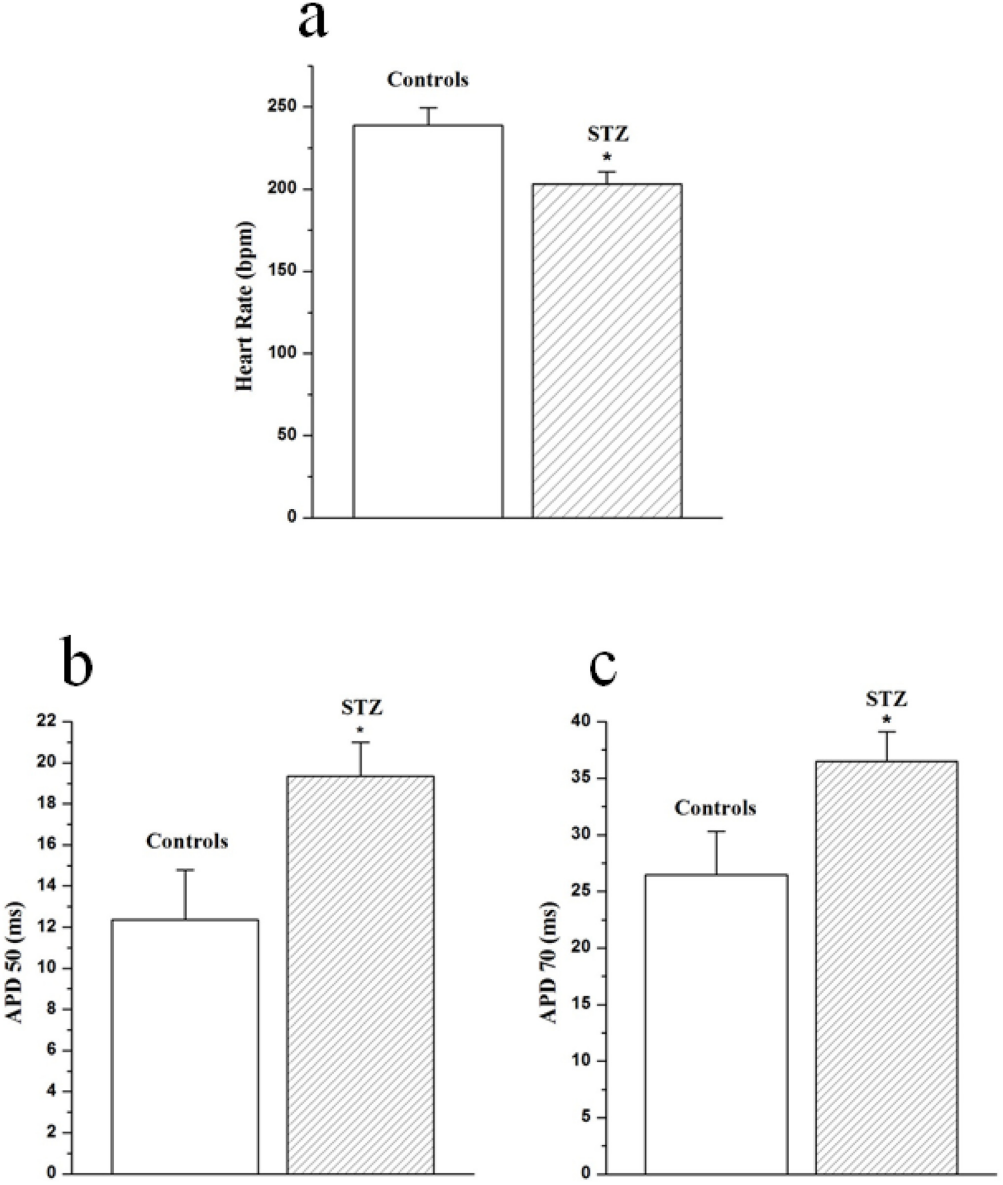
Effects of STZ-induced diabetes on heart rate (a), action potential duration at 50% (b) and 70% (c) recovery from peak action potential. Data are mean ± SEM, n = 12 hearts.

Heart rate and action potential duration data are shown in Fig 2. Heart rate was significantly (P<0.05) reduced in STZ (203±7 bpm, n = 12) compared to control (239±11 bpm, n = 12) heart (Fig 2a). Time to peak action potential was not significantly (P>0.05) altered in STZ (4.9±0.3 ms) compared to control (4.7±0.3 ms) heart. Action potential duration (APD) at 50% repolarization was significantly prolonged in STZ (19.3±1.7 ms) compared to control (12.4±2.4 ms) heart (Fig 2b). APD at 70% repolarization was also significantly prolonged in STZ (36.5±2.6 ms) compared to control (26.4±3.9 ms) heart (Fig 2c).

### Expression of mRNA

**Fig 3:**
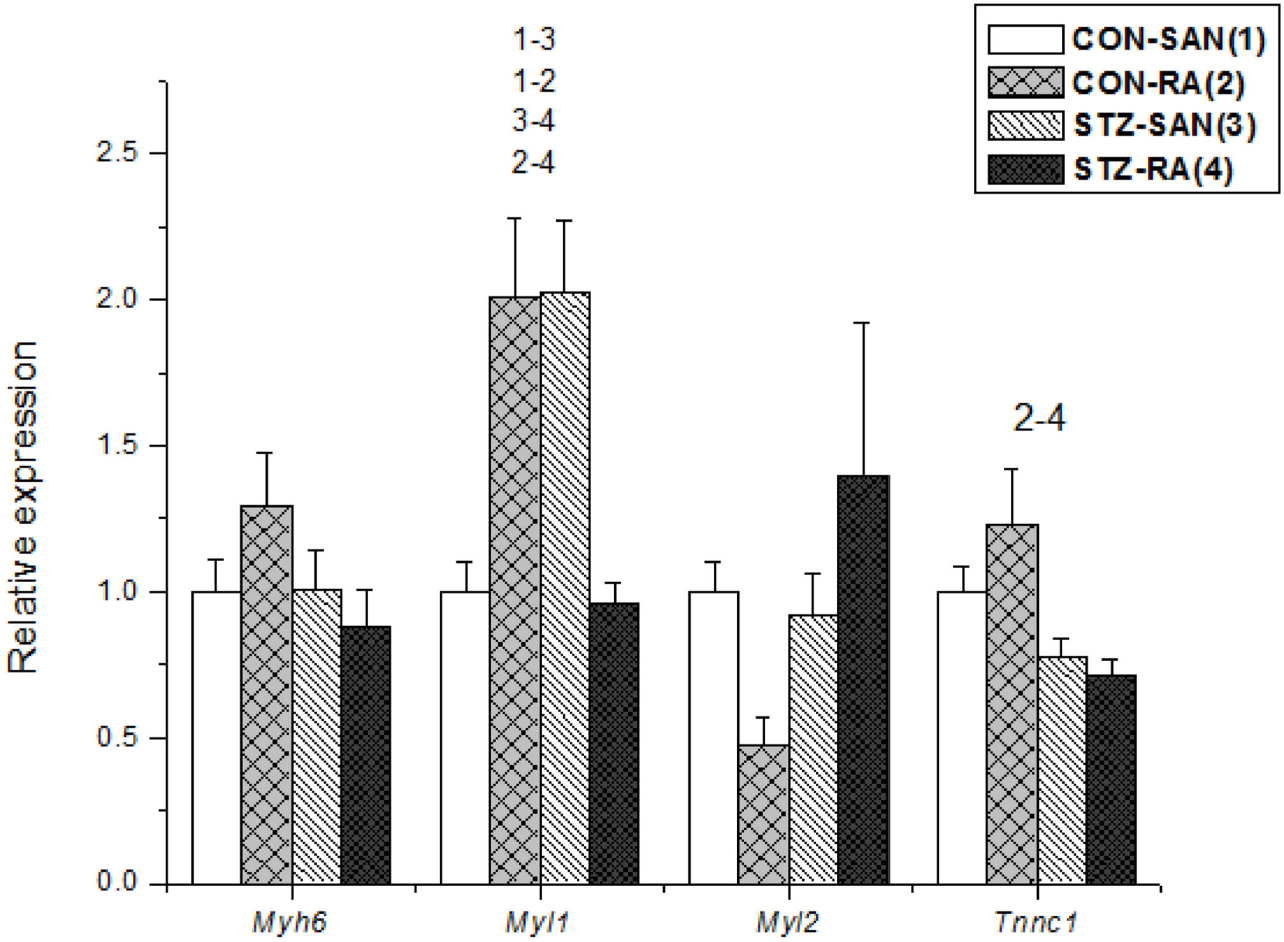
Expression of genes encoding various cardiac muscle proteins. Data are mean ± SEM, n = 4–10 samples from STZ and control rat each containing SANs from 2 hearts.

**Fig 4:**
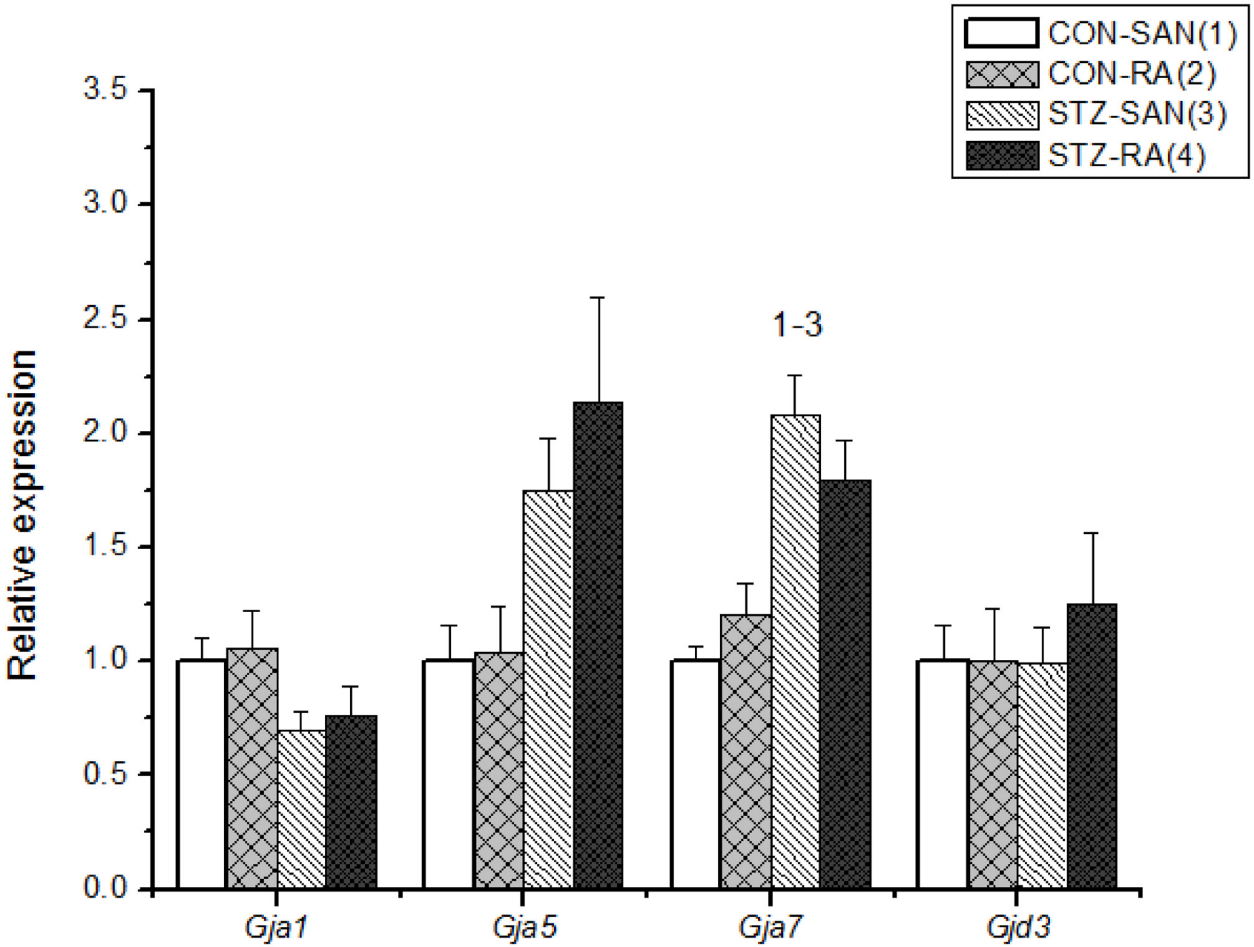
Expression of genes encoding various intercellular proteins. Data are mean ± SEM, n = 6–10 samples from STZ and control rat each containing SANs from 2 hearts.

**Fig 5:**
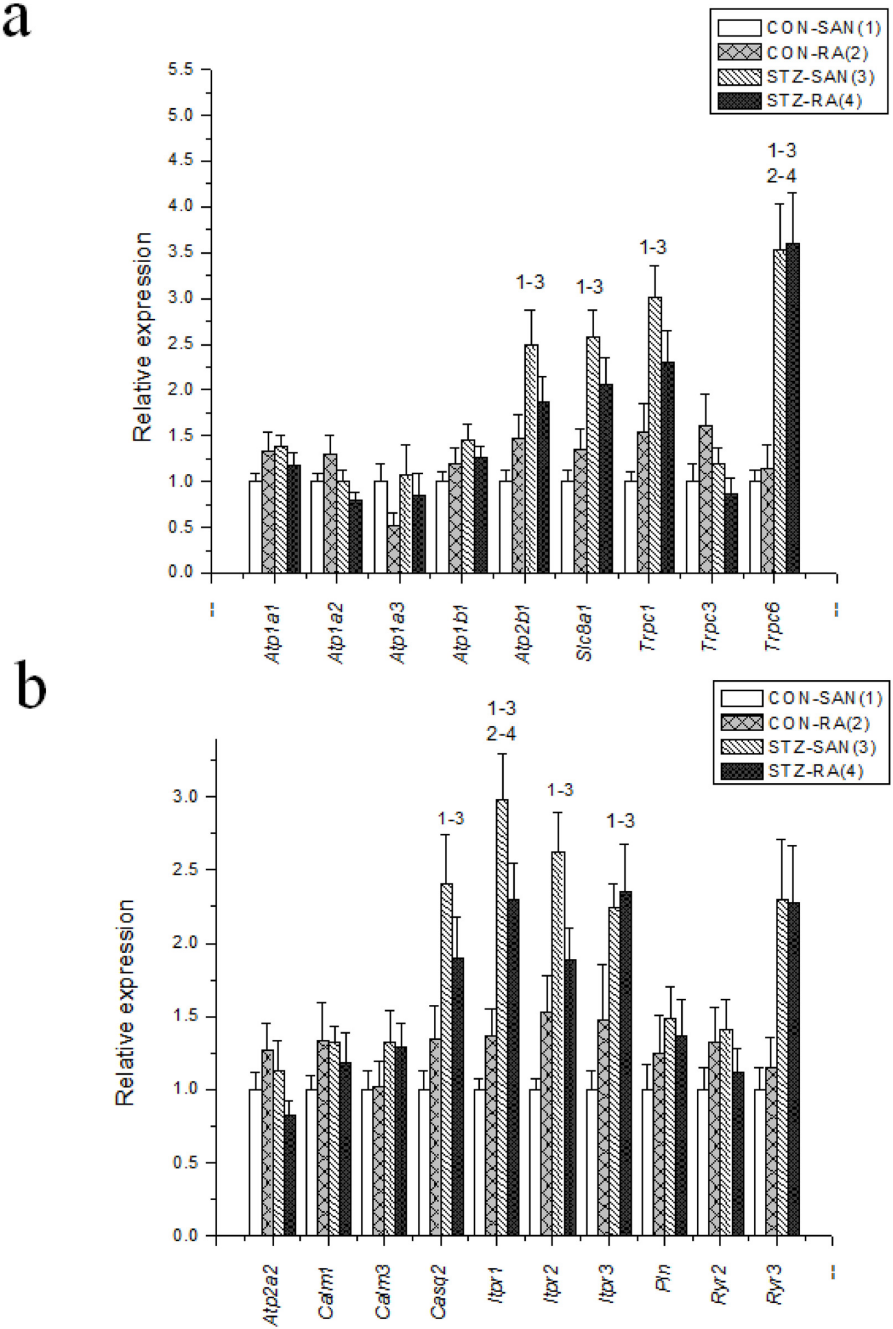
(a) Expression of genes encoding various intracellular Ca^2+-^transport and regulatory proteins and (b) membrane transport proteins. Data are mean ± SEM, n = 6–10 samples from STZ and control rat each containing SANs from 2 hearts.

**Fig 6:**
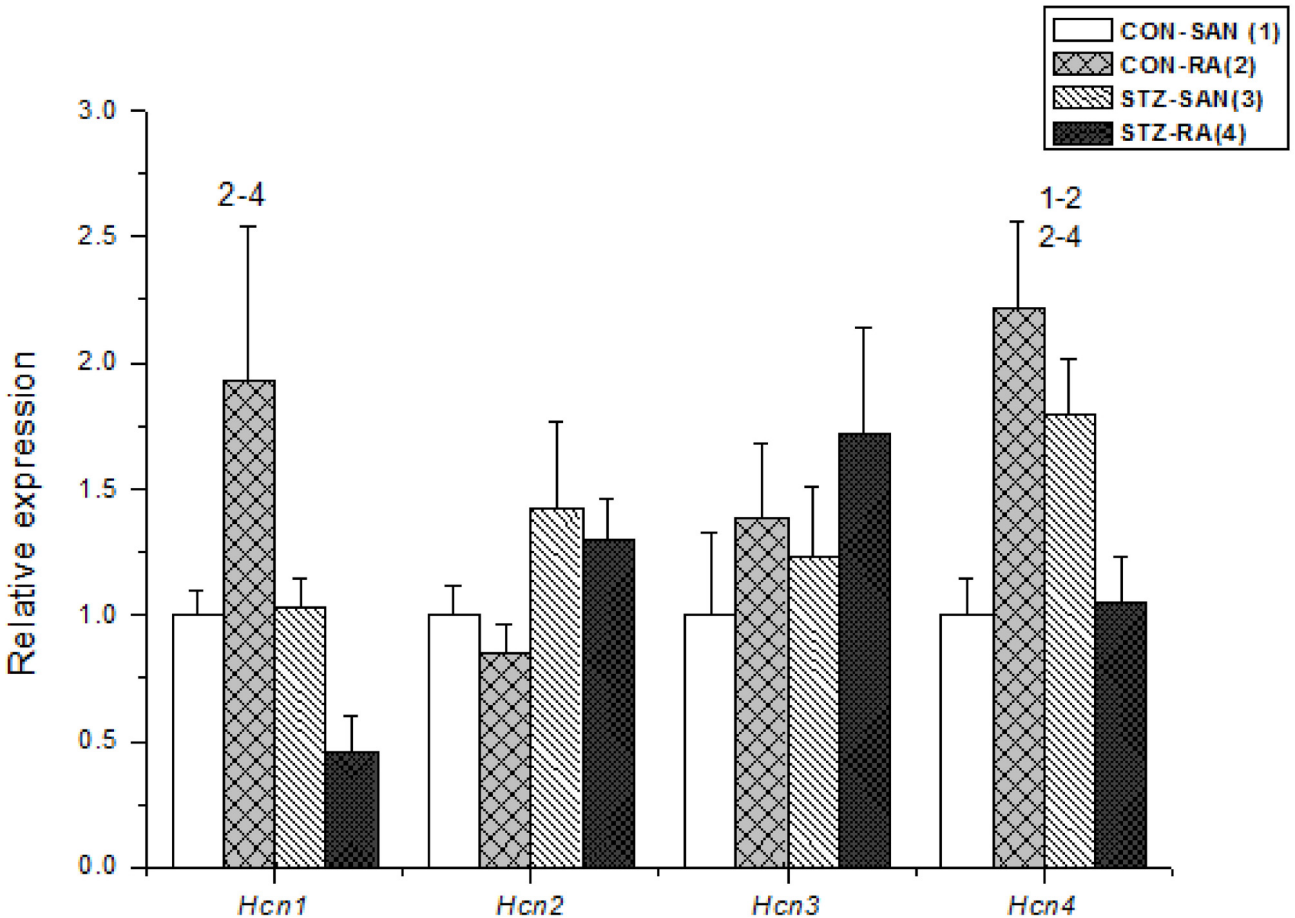
Expression of genes encoding various hyperpolarization-activated cyclic-nucleotide-gated channels. Data are mean ± SEM, n = 5–10 samples from STZ and control rat each containing SANs from 2 hearts.

**Fig 7:**
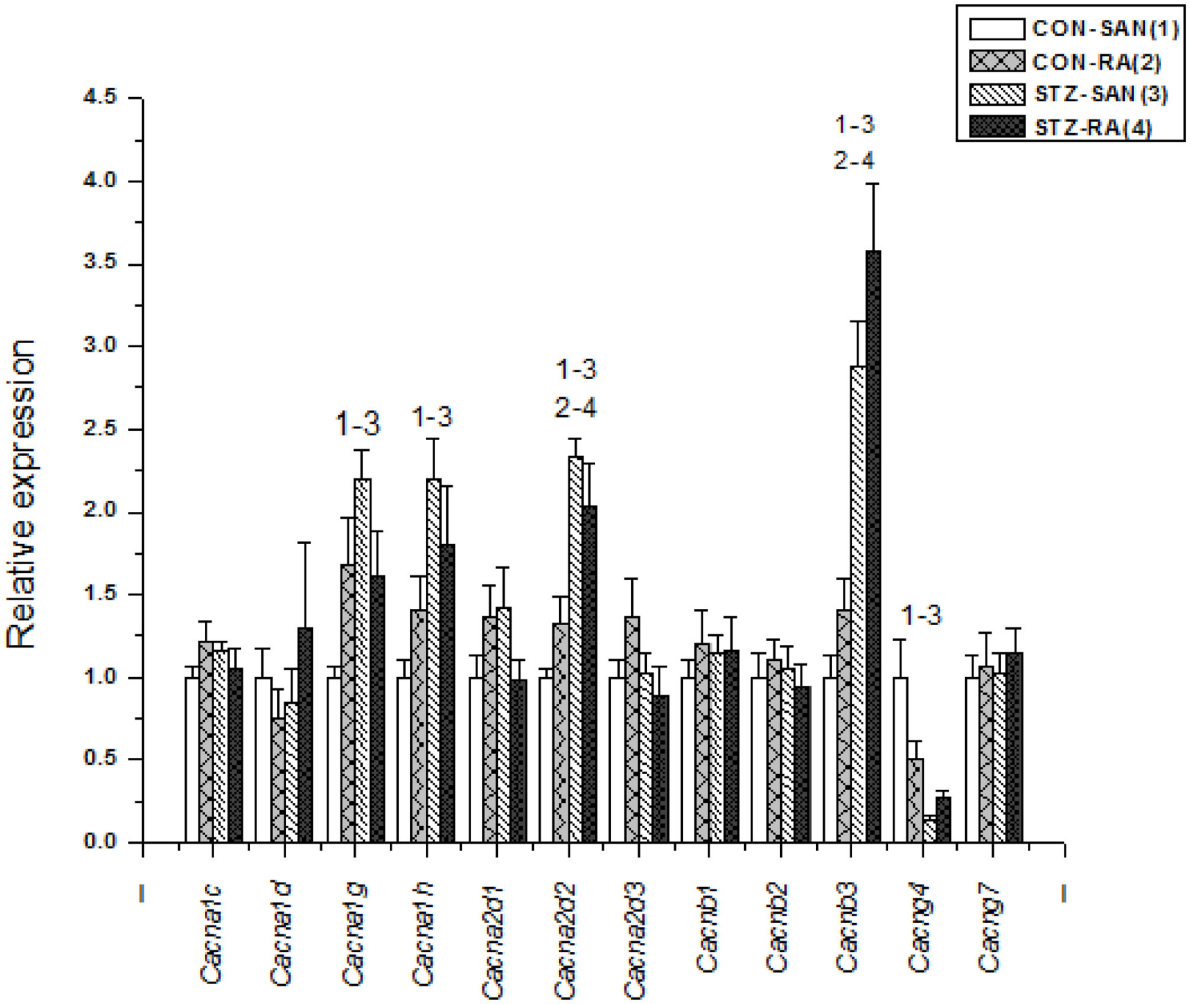
Expression of genes encoding various calcium channel proteins. Data are mean ± SEM, n = 5–10 samples from STZ and control rat each containing SANs from 2 hearts.

**Fig 8:**
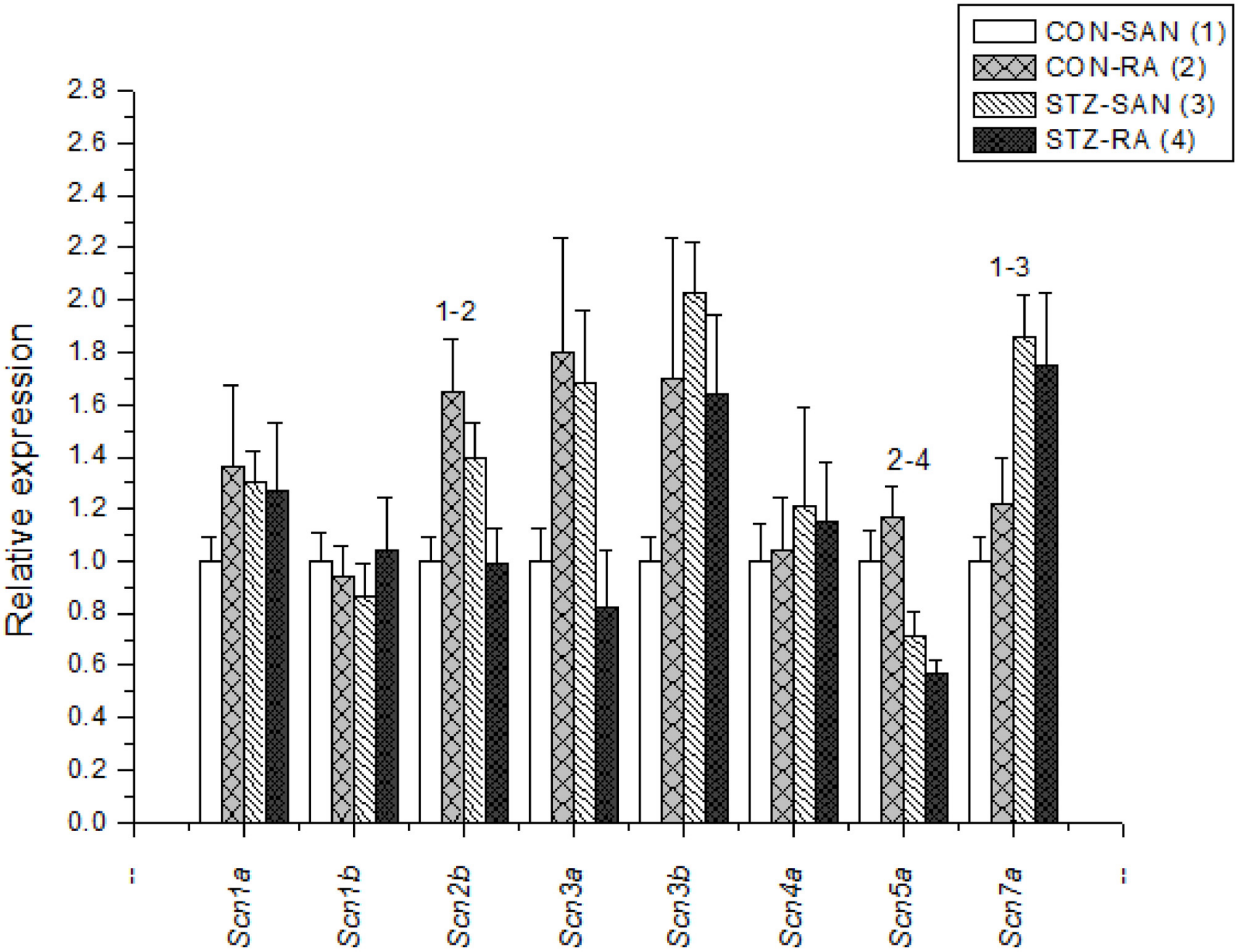
Expression of genes encoding various sodium channel proteins. Data are mean ± SEM, n = 8–10 samples from STZ and control rat each containing SANs from 2 hearts.

**Fig 9:**
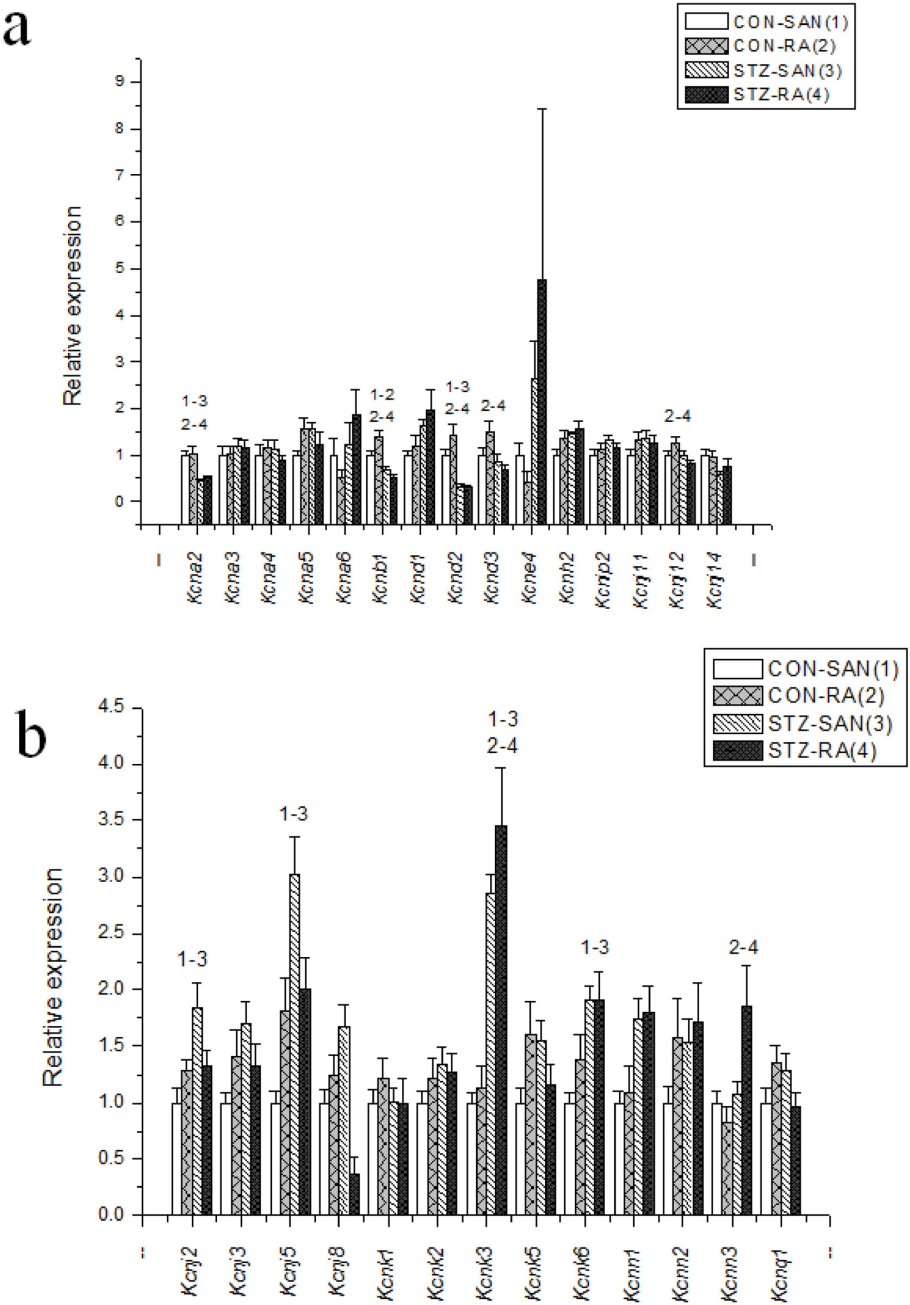
a and b—Expression of genes encoding various potassium channel proteins. Data are mean ± SEM, n = 6–10 samples from STZ and control rat each containing SANs from 2 hearts.

**Fig 10:**
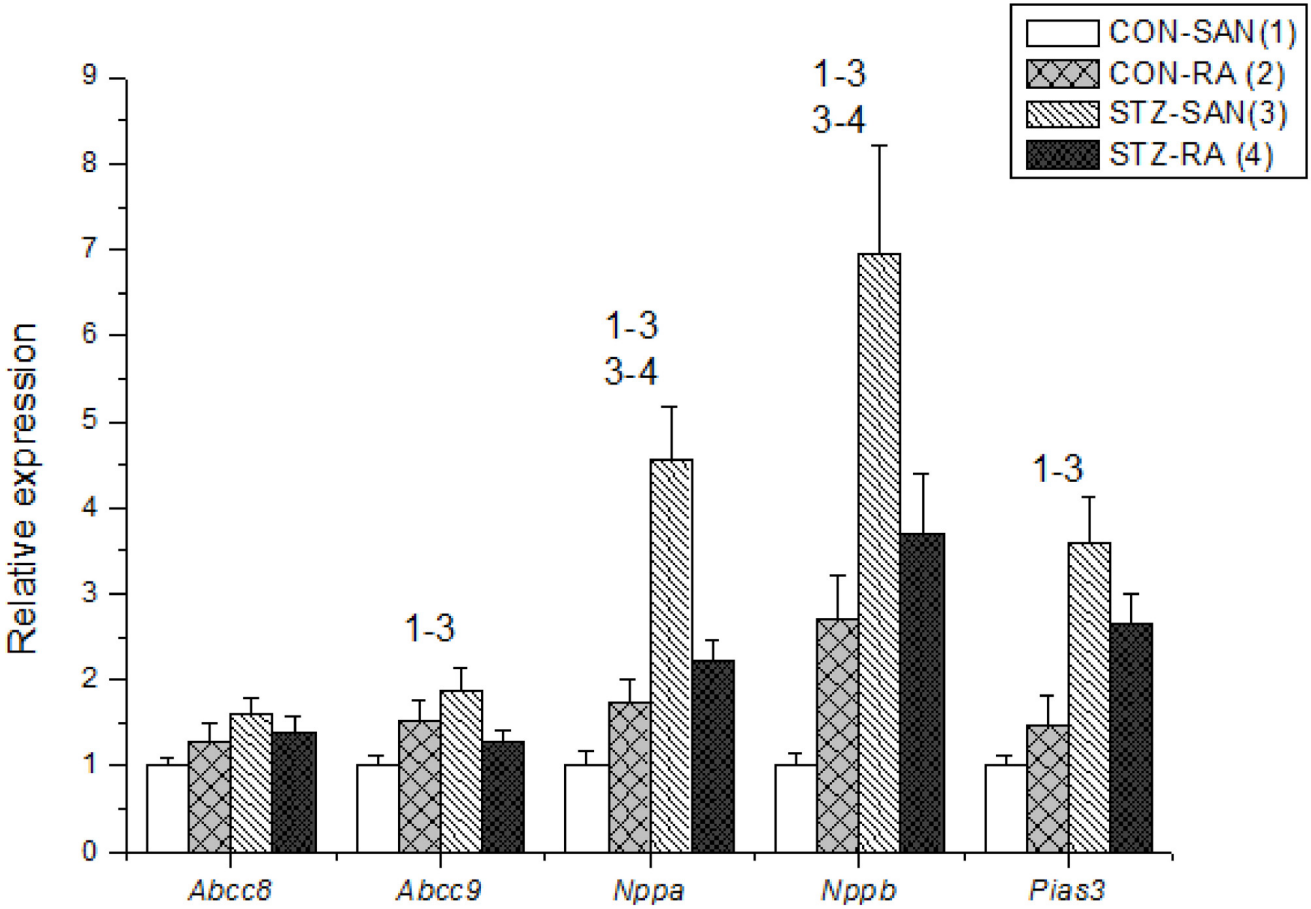
Expression of genes encoding miscellaneous cardiac proteins. Data are mean ± SEM, n = 8–10 samples from STZ and control rat each containing SANs from 2 hearts.

Expression of mRNA for cardiac muscle proteins are shown in Fig 3. Expression of *Myl1* was significantly (P<0.05) upregulated in STZ-SAN compared to CON-SAN whereas *Myl1* was significantly downregulated STZ-RA compared to CON-RA. Expression of mRNA for intercellular proteins are shown in Fig 4. Expression of *Gja7* was upregulated in STZ-SAN compared to CON-SAN. Expression of mRNA for cell membrane transport and intracellular Ca^2+^ transport are shown in Fig 5a and 5b, respectively. Among the cell membrane transport and Ca^2+^ transport proteins expression of *Atp2b1*, *Slc8a1*, *Trpc1*, *Trpc6*, *Casq2* and *Itpr1-3* were significantly upregulated and *Ryr3* was modestly upregulated in STZ-SAN compared to CON-SAN. Expression of mRNA for hyperpolarization-activated cyclic nucleotide-gated channel proteins are shown in Fig 6. *Hcn4* was modestly upregulated in STZ-SAN compared to CON-SAN however, the difference was not significant. Expression of mRNA for calcium channel proteins are shown in Fig 7. Expression of *Cacna1g*, *Cacna1h*, *Cacna2d* and *Cacnb3* were upregulated whilst *Cacng4* (7-fold) was downregulated in STZ-SAN compared to CON-SAN. Expression of mRNA for sodium channel proteins are shown in Fig 8. Expression of *Scn7a* was upregulated in STZ-SAN compared to CON-SAN. Expression of mRNA for potassium channel proteins are shown in Fig 9a and 9b. Expression of *Kcna2* and *Kcnd2* were downregulated whilst *Kcnj2*, *Kcnj5*, *Kcnk3* and *Kcnk6* were upregulated in STZ-SAN compared to CON-SAN. Expression of *Kcnd2*, *Kcnd3* and *Kcnj12* were downregulated and *Kcnk3* and *Kcnn3* were upregulated in STZ-RA compared to CON-RA. Expression of mRNA for miscellaneous cardiac proteins are shown in Fig 10. Expression of *Abcc9*, *Nppa*, *Nppb* and *Pias3* were upregulated in STZ-SAN compared to CON-SAN.

### Expression of protein

**Fig 11:**
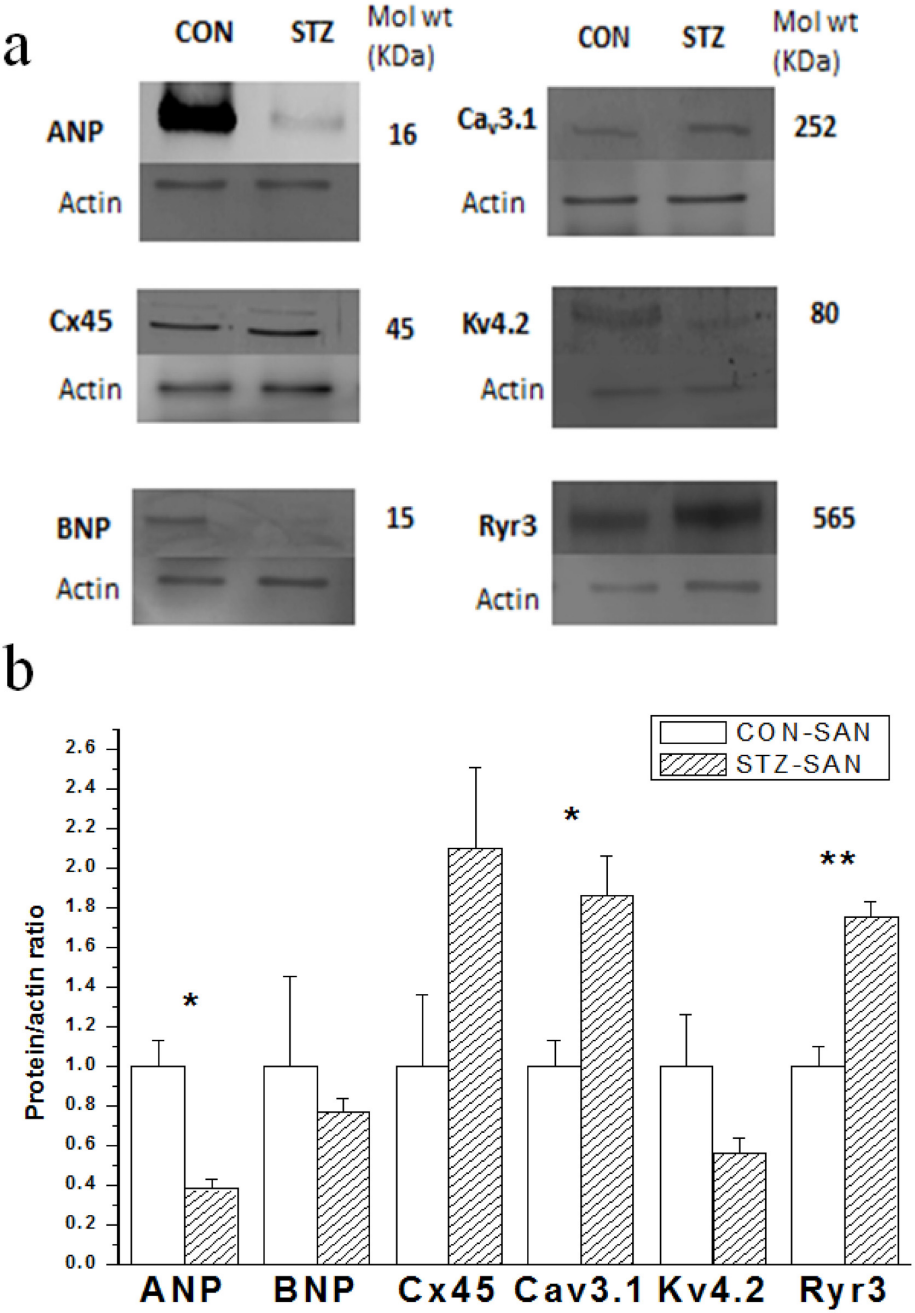
Typical Western blots comparing expression of various proteins from STZ and control SAN are shown in Fig 11a. The blots shown are representative of 3 individual samples from STZ and control rats each containing SANs from 2 hearts. The Protein/actin ratio for the different proteins are shown in Fig 11b. Data are mean ± SEM, n = 3 samples from STZ and control rat each containing SANs from 2 hearts. * P<0.05, ** P<0.01

Western blot techniques were used to compare expression of selected proteins in STZ and control SAN and typical results are shown in Fig 11a. Expression of ANP was significantly reduced whilst Cav3.1 and Ryr3 were increased in STZ compared to control SAN (Fig 11b).

## Discussion

Spontaneous heart rate was lower in STZ compared to control hearts. Previous *in vivo* biotelemetry experiments have also demonstrated reduced heart rate in STZ rat [5,21]. Prolongation of SAN action potentials in isolated perfused heart and prolonged pacemaker cycle length and sino-atrial conduction time in isolated sino-atrial preparations have been demonstrated in STZ rat heart [9,10]. Collectively, data from various isolated preparations suggest that the reduced heart rate in STZ rat could, at least in part, may be attributed to intrinsic defects in the electrophysiological function of the SAN. An early step in elucidating the mechanisms that underlie low heart rate in STZ rat is to study the profile of genes associated with proteins that are involved in the generation and propagation of the SAN action potential. Of particular interest were genes encoding *Gja7 (2-fold)*, *Trpc1 (3-fold)*, *Trpc6 (4-fold)*, Ryr3 (2-fold), *Cacna1g (2-fold)*, *Cacnb3 (3-fold)*, *Kcnj5 (3-fold)*, *Nppa* (5-fold) and *Nppb* (7-fold) which were upregulated and *Cacng4* (3-fold) which was downregulated in STZ-SAN compared to CON-SAN.

*Gja7* (Cx45) was upregulated in STZ-SAN compared to CON-SAN. Cx45 protein was also moderately increased in STZ-SAN compared to CON-SAN. Connexin proteins, pore forming subunits of gap junctions, play an important role in ensuring efficient cell-to-cell communication and the maintenance of cardiac rhythmicity [22]. At least five connexins (Cx30.2, Cx37, Cx40, Cx43 and Cx45) are prominently expressed in the heart and each shows regional and cell type specific expression [22,23]. *Gja7* (Cx45) is expressed in the SAN and previous studies have demonstrated upregulation of genes encoding connexin proteins, and in particular Cx45, in STZ-SAN [10,24–26]. It is known that action potential duration and SAN conduction and pacemaker cycle length can be prolonged in diabetic heart and this may be associated with remodeling of connexin proteins [10,11].

*Trpc1* (TRPC1) and *Trpc6* (TRPC6) were upregulated in STZ-SAN compared to CON-SAN. The transient receptor potential (TRP) channels are a large family of non-selective and non-voltage gated ion channels that are widely expressed in human tissue including the heart and vasculature [27,28]. TRPC1 and TRPC6 are mechano-sensitive, non-selective cation channels that are expressed in mouse ventricular muscle [29]. In the cardiovascular system the TRPC family has been found to play a role in vascular and cardiac disease [30]. Upregulation of TRPC channels is involved in the development of cardiac hypertrophy and heart failure [27,31,32]. TRPC6 is induced in heart hypertrophy and inhibition of TRPC6 has been shown to suppress agonist-induced hypertrophic responses [33–35]. Whilst the physiological role of the TRP channels in the SAN is unclear, upregulation of *Trpc1* and *Trpc6* in STZ-SAN if associated with altered entry of Na^+^ or Ca^2+^ through non-selective TRP channels might have implications for the generation of the pacemaker or action potential in diabetic SAN cells [31,36,37].

*Cacna1g* (Ca_v_3.1) was upregulated in STZ-SAN compared to CON-SAN however, expression of *Cacna1g* was not altered in STZ-RA compared to CON-RA. Ca_v_3.1 protein was also increased in STZ-SAN compared to CON-SAN. Previous studies have reported upregulation of *Cacna1g* in ventricle from the Goto-Kakizaki rat, an experimental model of type 2 diabetes mellitus, and the Zucker diabetic fatty rat [38,39]. The protein encoded by *Cacna1g* represents the alpha 1G subunit, also known as Ca_v_3.1, of the T-type calcium channel. In the heart T-type Ca^2+^ channels are found in the SAN and conduction cells [40]. In mice disruption of the gene encoding Ca_v_3.1channels abolishes T-type Ca^2+^ current in isolated cells from the SAN and atrioventricular node without affecting L-type Ca^2+^ current. Inactivation of *Cacna1g* slowed the heart rate *in vivo* and prolonged the SAN recovery time and slowed pacemaker activity of individual SAN cells through a reduction of the slope of the diastolic depolarization [41]. Upregulation of *Cacna1g* (Ca_v_3.1) might be expected to increase T-type Ca^2+^ current and hence, the slope of the pacemaker potential and heart rate in STZ rat.

*Cacnb3* (Ca_v_β3) was upregulated in STZ-SAN compared to CON-SAN. *Cacnb3* (Ca_v_β3) encodes expression of the beta 3 subunit (Ca_v_β3) of the L-type Ca^2+^ channel. Previous studies have demonstrated that overexpression of the beta subunits (1–4) in adult cultured heart cells increased whole-cell L-type Ca^2+^ current density [42]. In the SAN L-type Ca^2+^ current contributes to the pacemaker potential and generates the upstroke of the action potential therefore upregulation of *Cacnb3* (Ca_v_β3) might be expected to increase L-type Ca^2+^ current, the slope of the pacemaker potential and hence heart rate.

*Ryr3* (RYR3) was upregulated in STZ-SAN compared to CON-SAN. RY3 protein was also increased in STZ-SAN compared to CON-SAN. Various studies have shown that sub-sarcolemmal diastolic Ca^2+^ release from the sarcoplasmic reticulum (SR) may contribute to the generation of electrical activity in SAN cells [43,44]. Upregulation of the SR Ca^2+^ release channel (ryandodine receptor) would be expected to facilitate increased release of Ca^2+^ from the SR and hence, might influence spontaneous electrical activity in the SAN cell.

*Cacng4* (Ca_v_γ4) was downregulated (3-fold) in STZ-SAN compared to CON-SAN however, expression of *Cacng4* was not altered in STZ-RA compared to CON-RA. The cardiac voltage-gated L-type Ca^2+^ channel is the Ca^2+^ channel that is required for excitation-contraction coupling and that also contributes to the plateau phase of the cardiac action potential and pacemaker activity in nodal cells [45,46]. The protein encoded by *Cacng4* (Ca_v_γ4) represents one of the gamma subunits of the L-type Ca^2+^ channel. It has been reported that the gamma subunits expressed in heart (gamma 4, 6, 7 and 8) form macromolecular complexes with Cav1.2 and can differentially modulate its function [45]. Downregulation of *Cacng4* might have implications for the modulation and hence, function of the L-type Ca^2+^ channel and L-type Ca^2+^ current which in turn are important for generation of the pacemaker potential and the upstroke of the SAN action potential.

*Nppa* (ANP) and *Nppb* (BNP) were upregulated in STZ-SAN compared to CON-SAN however, neither *Nppa* or *Nppb* were significantly altered in STZ-RA compared to CON-RA. ANP protein was also significantly reduced in STZ-SAN compared to CON-SAN. Natriuretic peptides are a family of related peptides that include atrial natriuretic peptide (ANP) and B-type natriuretic peptide (BNP) that are secreted from the cardiac atria and ventricles [47]. ANP and BNP decrease blood pressure and cardiac hypertrophy and BNP acts locally to reduce ventricular fibrosis and they are both involved in the pathogenic mechanisms leading to major cardiovascular diseases, including heart failure, coronary heart diseases, hypertension and left ventricular hypertrophy [47–49]. Previous studies have demonstrated the expression of ANP, albeit at low levels, in the SAN node [50,51]. ANP plays a key role in cardiac electrophysiology, modulating the autonomic nervous system and regulating the function of various cardiac ion channels [52]. Previous studies have demonstrated increases in ANP and BNP in blood plasma and atrial tissues and varying effects of ANP and BNP on the amplitude and kinetics of shortening and [Ca^2+^]_i_ in ventricular myocytes from STZ-induced diabetic rat [53,54]. BNP has been shown to increase heart rate and electrical conduction velocity in isolated hearts and in the SAN and also increase spontaneous action potential frequency in isolated SAN myocytes [55–57]. Upregulation of *Nppa* and *Nppb* in the SAN may be associated with mechanisms that compensate for the low heart rate seen in the STZ-induced diabetic heart [53,54]. Interestingly, expression of *Nppa* and *Nppb* were increased whilst ANP protein was reduced in STZ-SAN compared to CON-SAN. Previous studies have demonstrated increased ANP and BNP levels in plasma and atria in STZ rats compared to controls [53,54].

Collectively, this study has demonstrated differences in the profile of mRNA encoding a variety of proteins that are associated with the generation, conduction and regulation of electrical signals in the SAN of STZ-induced diabetic rat heart. Data from this study will provide a basis for a substantial range of studies to investigate whether these changes in mRNA translate into changes in electrophysiological function.
